# Exploring the health literacy status of people with hearing impairment: a systematic review

**DOI:** 10.1186/s13690-023-01216-x

**Published:** 2023-11-22

**Authors:** Zhaoyan Piao, Hanbin Lee, Yeongrok Mun, Hankil Lee, Euna Han

**Affiliations:** 1 College of Pharmacy, Yonsei Institute of Pharmaceutical Sciences, Yonsei University, 162-1 Songdo-Dong, Yeonsu-Gu, Incheon, Republic of Korea; 2 College of Pharmacy, Ajou University, Suwon, Gyeonggi-Do Republic of Korea

**Keywords:** Health literacy, Hearing impairment, Systematic review, Bias risk

## Abstract

**Background:**

People with hearing impairment have many problems with healthcare use, which is associated with health literacy. Research on health literacy is less focused on people with hearing impairments. This research aimed to explore the levels of health literacy in people with hearing impairment, find the barriers to health literacy, and summarize methods for improving health literacy.

**Methods:**

A systematic review was conducted using three databases (PubMed, Cochrane, and Embase) to search the relevant articles and analyze them. The studies were selected using pre-defined inclusion/exclusion criteria in two steps: first, selection by examining the title and abstract; and second, after reading the study in full. The Risk of Bias Assessment Tool for Nonrandomized Studies (RoBANS) was used to assess the quality of the articles.

**Results:**

Twenty-nine studies were synthesized qualitatively. Individuals with hearing impairment were found to have lower health literacy, when compared to those without impairment, which can lead to a higher medical cost. Most of the people with hearing impairment faced barriers to obtaining health-related information and found it difficult to communicate with healthcare providers. To improve their health literacy, it is essential to explore new ways of accessing health information and improving the relationship between patients and healthcare providers.

**Conclusions:**

Our findings show that people with hearing impairment have lower health literacy than those without. This suggests that developing new technology and policies for people with hearing impairment is necessary not to mention promoting provision of information via sign language.

**Trial registration:**

OSF: https://doi.org/10.17605/OSF.IO/V6UGW. PROSPERO ID: CRD42023395556.

**Supplementary Information:**

The online version contains supplementary material available at 10.1186/s13690-023-01216-x.

## Introduction

The term *health literacy* has come to represent a variety of meanings as research in related fields intensified [1]. “The cognitive and social skills that determine the individuals’ motivation and ability to gain access to, understand, and use information in ways that promote and maintain good health” is referred to as health literacy in the second edition of the WHO Health Promotion Glossary [2] published in 1998. In 1999, the American Medical Association’s Ad Hoc Committee on Health Literacy [3], defined health literacy as a “constellation of skills, including the ability to perform basic reading and numerical tasks required to function in the health care environment,” including the “ability to read and comprehend prescription bottles, appointment slips, and other essential health-related materials.” The Institute of Medicine (IOM) [4] decided to use the definition adopted by Healthy People 2010 [5]thus: “the degree to which individuals have the capacity to obtain, process, and understand basic health information and services needed to make appropriate health decisions” [6]. This concept includes many scenarios in which individuals may encounter and interact with health concerns; nonetheless, all the above-mentioned definitions attempt to characterize health literacy as a problem of individual ability and competence [4].

Health literacy is influenced by many factors, including social and personal factors [7], while indirectly impacting health outcomes by changing the status of the relationship between health care providers and patients. Hearing is a factor that influences the level of health literacy, which is important for accessing, understanding, judging, and using health information appropriately. Therefore, it is necessary to objectively explore the association between health literacy and hearing impairment to achieve better health outcomes among people with hearing impairment. Previous reviews looked into the capacity of cancer education to promote health literacy in patients with hearing difficulties [8, 9], but the focus was confined to cancer-related health literacy. In our study, health literacy was not limited to cancer-related but also to other health domains, and we summarized the methods that can improve health literacy except education. In this study, we conducted a comprehensive literature review on the health literacy of people with hearing impairment to understand the level and status of health literacy in persons with hearing loss, outline the challenges and concerns of individuals with hearing disabilities, and propose strategies to improve health information transmission.

## Methods

### Search strategy and data source

We conducted the systematic review according to the guidelines of the Preferred Reporting Items for Systematic Reviews and Meta-analysis (PRISMA). We extracted, analyzed, and integrated research papers found suitable as per the selection criteria. After searching three global search engines, we found there was no literature about both health literacy and hearing disability before 2000, the data search and analysis on research papers published in international journals were conducted from January 2000 to December 2021. We used three global search engines—including PubMed, Cochrane Library, and Embase Emtree—and MeSH (Medical Subject Headings) terms. The applicability of the subject terms for this search is only guaranteed to be valid until July 5, 2022—Additional file 1 describes the specific literature search methods in the three databases.

### Inclusion and exclusion criteria

Of the retrieved papers, duplicates were excluded using Endnote 20.3 (Bld 16073). We selected the corresponding literature using key questions prepared in the form of Participants, Interventions, Comparisons, Outcomes, Timing, Settings, Study Design (PICOTS-SD)—that added timing, settings, and study design to the PICO (Participants, Intervention, Comparison, Outcome) framework [10]. The key questions used are listed in Additional file 2. Two authors independently screened papers for relevance to this study by applying the literature selection and exclusion criteria described in Table 1. We selected studies that were relevant both to people with hearing impairment and health literacy and published between 2000 and 2021. Meanwhile, we had five exclusion criteria, including requirements for the topic of the study, population, year of publication, language of publication, and originality. We excluded papers using title and abstract and added manual searches using single specific terms, such as "hearing disability," to find relevant articles.

**Table 1:** Inclusion and exclusion criteria

Inclusion criteria	− The study was conducted on people with hearing impairment; − Studies reporting levels of health literacy, influencing factors, measurement methods or health care outcomes; − Studies in which the control group was non-disabled, or studies conducted on people with hearing disabilities alone; − Studies conducted after 2000
Exclusion criteria	− Research not related to the topic; − Studies not simultaneously related to people with hearing impairment and health literacy; − Studies not conducted after 2000; − Non-original research; − Articles not written in Korean or English

### Data extraction

**Fig. 1:**
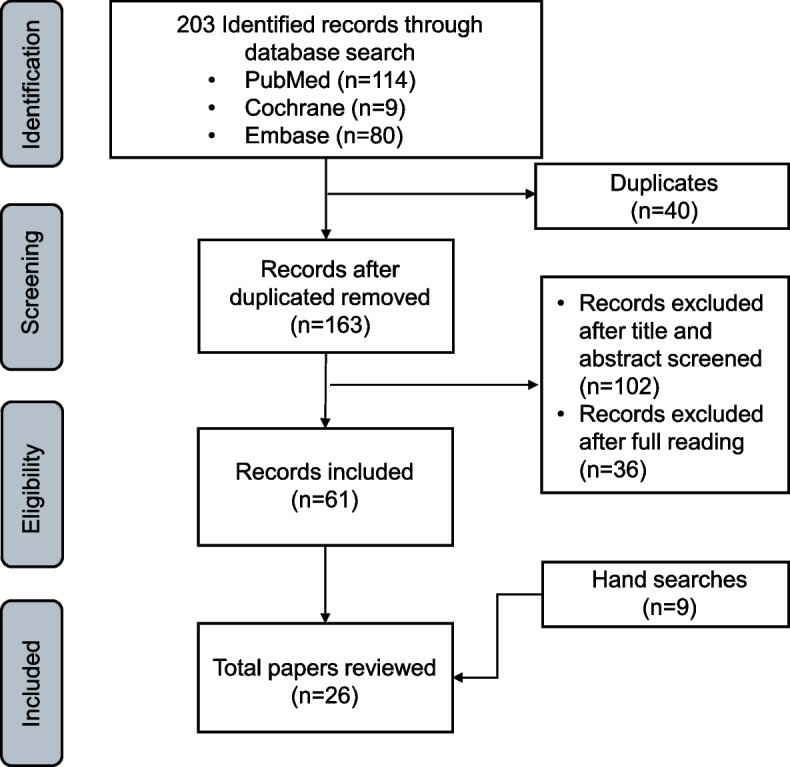
PRISMA flow diagram

As shown in the Figure, we initially gathered 163 publications after deleting duplicates. After further exclusion by abstract, title, and full text, 17 articles remained, and with the addition of 12 articles from the manual search, 29 articles were finally selected for the study (Fig. 1).

### Assessment of the risk of bias for the studies

We used the Risk of Bias Assessment Tool for Nonrandomized Studies (RoBANS) to evaluate the quality of the selected studies. RoBANS contains six domains, including selection of participants, confounding variables, measurement of exposure, blinding of the outcome assessments, incomplete outcome data, and selective outcome reporting. For each domain, the bias risk was classified as “low,” “high,” or “unclear” [11]. According to Kim et al. [11], the total bias is evaluated by three relatively more important domains (selection of participants, confounding variables, and incomplete outcome data) out of the six, and if more than two of the three have the same bias—such as “low”—then the total bias is “low.” If the three domains have different biases, then the total bias is “unclear.” Assessment of bias was evaluated by two authors individually, but in case of disagreement, we invited another author to co [12, 13].

### Analysis method

Due to the diversity of the literature in terms of populations, settings, interventions, and outcomes, it was not possible to consolidate the results quantitatively. Alternatively, we collated the main components of the literature and analyzed the key themes between the selected papers, thus establishing commonalities and differences. The papers were collated in terms of countries, characteristics of subjects, study aims, methodology, main findings, and tools for measure health literacy. The results on health literacy status were separately collated for the characteristics of health literacy problems, difference in health literacy levels among people with hearing disabilities, barriers against health literacy, and the ways of improving health literacy.

The final collected papers were categorized into quantitative and qualitative research based on the research method used. Taking the unknown into consideration, the research was classified as quantitative if objective generalized results could be obtained using health literacy tests. If observation and interview methods were conducted to investigate the phenomenon, the research was classified as qualitative.

This study collated the results of the research based on the following. First, the characteristics of the included studies were examined. Second, what is the level of health literacy for people with hearing impairment compared to those without such impairment? Third, what are the barriers to health literacy for people with hearing impairment? Fourth, how does the level of health literacy for the hearing-impaired lead to health management? Fifth, what are the ways to improve the health literacy of people with hearing impairment?

## Results

### Characteristics of included studies and results of bias assessment

In general, most of our selected papers had low bias and were of high quality. As shown in Additional file 3, the risk of bias was rated as “low” for 25 of the 29 papers, “high” for 2 papers, and “uncertain” for 2 papers. Twenty of the 26 papers were from the United States, one involved both Kuwait and Saudi Arabia, one was from both South Africa and China, and the other seven from Canada, South Africa, Germany, Turkey, Nigeria, Australia, and Swaziland. The sample size varied from 7 to 19,223 people, with 24 studies having fewer than 1,000 and 4 papers having more than 10,000. There were 22 quantitative studies and 7 qualitative studies. Four of the studies involved teenagers, while the others were conducted on adults. Four of the studies included just female hearing-impaired participants, while none involved only male hearing-impaired participants. Twenty-two studies were conducted by survey, three by interview, and four by focus groups (Table 2).

**Table 2:** Characteristics of study included

Study(year)	Country	Title	Methodology	N	Population	Measurement tool
Gregg [14] (2002)	United States	Designing a Curriculum on Internet Health Resources for Deaf High School Students	Survey (qualitative)	22	Deaf high school students	Questionnaire to investigate the level of Internet skills
Steinberg [15] (2006)	United States	Health Care System Accessibility: Experiences and Perceptions of Deaf People	Focus group (qualitative)	91	91 adults with hearing disabilities (aged 24–83 years) who predominantly communicate in American Sign Language	None
Groce [16] (2006)	Swaziland	HIV/AIDS and Disability: A Pilot Survey of HIV/AIDS Knowledge Among a Deaf Population in Swaziland	Survey (quantitative)	191	91 hearing impaired and 100 hearing adults	Questionnaire related to HIV/AIDS
Groce [17] (2007)	Nigeria	HIV/AIDS and Disability: Differences in HIV/AIDS Knowledge Between Deaf and Hearing People in Nigeria	Survey (quantitative)	100	50 teenagers with hearing impairment and 50 hearing adults	Questionnaire related to HIV/AIDS
Choe [18] (2009)	United States	The Impact of Cervical Cancer Education for Deaf Women Using a Video Educational Tool Employing American Sign Language, Open Captioning, and Graphics	Survey (quantitative)	130	Women with hearing impairment (aged 19–93)	Questionnaire related to cervical cancer
Pollard [19] (2009)	United States	Health-Related Vocabulary Knowledge Among Deaf Adults	Survey (quantitative)	57	People with hearing loss (aged 21–67 years) participated, with 27 women and 29 men	REALM
Hoang [20] (2011)	United States	Assessing Deaf Cultural Competency of Physicians and Medical Students	Survey (qualitative)	1445	640 non-DCT students, 25 DCT students, and 780 medical school instructors	Questionnaires related to deaf culture and clinical dilemmas of deaf people
Convery [21] (2011)	Australia	Management of Hearing Aid Assembly by Urban-Dwelling Hearing-Impaired Adults in a Developed Country: Implications for a Self-Fitting Hearing Aid	Survey (quantitative)	80	80 participants aged 45–90 years with hearing impairment	S-TOFHLA
Maddalena [22] (2012)	Canada	Palliative and End-of-Life Care in Newfoundland’s Deaf Community	Interview (qualitative)	7	Members of the Deaf community, residents of the Northeast Avalon Region, and the caregiver of a deceased person with disabilities (aged 40–65 years)	None
Yao [23] (2012)	United States	Cervical Cancer Control: Deaf and Hearing Women’s Response to an Educational Video	Survey (quantitative)	133	127 hearing loss women (aged 19–93 years) and 106 hearing women (aged 18–85 years)	Questionnaire related to cervical cancer
Berman [24] (2013)	United States	Breast Cancer Knowledge and Practices Among Deaf Women	Interview (quantitative)	209	209 hearing loss women (aged 40 years and up) with poor levels of education	Selected questions from questionnaires previously applied to people with hearing impairment and from a standard survey instrument, as well as from a pool of items used in other cancer prevention and control studies in low-literacy, cultural, and linguistic populations
Jensen [25] (2013)	United States	Ovarian Cancer: Deaf and Hearing Women’s Knowledge Before and After an Educational Video	Survey (quantitative)	107	55 hearing loss women (aged 18 years and up) and 52 hearing women (aged 18 years and up)	Questionnaire related to ovarian cancer
Convery [26] (2013)	South Africa, China	Hearing-aid Assembly Management Among Adults from Culturally and Linguistically Diverse Backgrounds: Toward the Feasibility of Self-Fitting Hearing Aids	Survey (quantitative)	80	40 hearing-impaired South Africans and 40 Chinese individuals (aged 32–92 years)	S-TOFHLA
Mckee [27] (2015)	United States	Assessing Health Literacy in Deaf American Sign Language Users	Interview (quantitative)	405	166 adults with hearing loss (aged 40–70 years) and 239 hearing people (aged 40–70 years)	Transform the New Vital Sign (NVS) tool into a sign language interface
Ferguson [28] (2015)	United States	Communication Needs of Patients with Altered Hearing Ability: Informing Pharmacists’ Patient Care Services Through Focus Groups	Focus group (qualitative)	20	Patients with hearing loss (aged 18 years and up) using American Sign Language who have more than two long-term prescription medications	None
Smith [29] (2015)	United States	Deaf Adolescents’ Learning of Cardiovascular Health Information: Sources and Access Challenges	Interview (qualitative)	20	Teenagers with hearing impairment using sign language (aged 9–12 years) from Rochester, New York	None
Kushalnagar [30] (2015)	United States	Health Websites: Accessibility and Usability for American Sign Language Users	Survey (quantitative)	32	Students with hearing impairment (aged 18 years and older) who know ASL	S-TOFHLA
Smith [31] (2016)	United States	Dimensions of Deaf/Hard-of-Hearing and Hearing Adolescents’ Health Literacy and Health Knowledge	Survey (quantitative)	281	187 D/HH and 94 hearing college-bound high school students	HLSI-SF S-TOFHLA CHDKQ
Haricharan [32] (2017)	South Africa	Health Promotion via SMS Improves Hypertension Knowledge for Deaf South Africans	Focus group (quantitative)	41	People with hearing disabilities in Cape Town (aged 18 years and up)	30 objective questionnaires to measure the effectiveness of SMS campaigns
Palmer [33] (2017)	United States	Bilingual Approach to Online Cancer Genetics Education for Deaf American Sign Language users Produces Greater Knowledge and Confidence than English Text Only: A Randomized Study	Survey (quantitative)	150	ASL users with hearing impairment (aged 18 years and up) with computer and internet access took part	Added items to the existing cancer genetics knowledge questionnaire
Kushalnagar [34] (2017)	United States	Critical Health Literacy in American Deaf College Students	Survey (quantitative)	38	Undergraduate students with hearing loss who use ASL (aged 22–38 years)	S-TOFHLA
Pinilla [35] (2019)	Germany	Primary Non-Communicable Disease Prevention and Communication Barriers of Deaf Sign Language Users: A Qualitative Study	Interview (qualitative)	15	Two had a history of diabetes, whereas 13 did not. All were members of the deaf community located in Germany	None
Stevens [36] (2019)	United States	Communication and Healthcare: Self-Reports of People with Hearing Loss in Primary Care Settings	Survey (quantitative)	1581	The survey information was distributed to the Hearing Loss Association of America email listserv; 1,682 people visited the survey link and 1,581 people responded	Survey questions developed by subject matter experts in hearing health
Gur [37] (2020)	Turkey	Health Literacy of Hearing-Impaired Adolescents, Barriers, and Misunderstandings they Encounter, and their Expectations	Survey (quantitative)	88	Students enrolling in a deaf or hard of hearing (D/HoH) vocational high school (aged 15–18 years)	TSY-32 based on HLS-EU
Tolisano [38] (2020)	United States	Can You Hear Me Now? The Impact of Hearing Loss on Patient Health Literacy	Survey (quantitative)	300	Adult patients (aged 18 years and up)	BHLS
Wells [39] (2020)	United States	Limited Health Literacy and Hearing Loss Among Older Adults	Telephone survey (quantitative)	19,223	Adults (aged 65 years and up)	The following question was used to test health literacy: “How confident are you filling out medical forms by yourself?”
Willink [40] (2020)	United States	Understanding Medicare: Hearing Loss and Health Literacy	Survey (quantitative)	10,510	With survey weights applied, the studied Medicare beneficiaries represented 50,084,169 beneficiaries	8 questions about health literacy
Almusawi [41] (2021)	Kuwait, Saudi Arabia	Disparities in Health Literacy During the COVID-19 Pandemic Between the Hearing and Deaf Communities	Survey (quantitative)	110	40 individuals with hearing impairment and 70 hearing adults	Questionnaire developed in relation to COVID-19
Tran [42] (2021)	United States	Health Literacy and Hearing Healthcare Use	Survey (quantitative)	1376	Between August 2018 and October 2019; adult patients (aged 18 years and older) who received hearing testing and finished a health literacy survey	BHLS

### Tools for measuring health literacy

The most used assessment tools in the articles we searched were the Rapid Estimate of Adult Literacy in Medicine (REALM), Test of Functional Health Literacy in Adults (TOFHLA), Newest Vital Sign (NVS), Health Literacy Skills Instrument (HLSI), European Health Literacy Survey (HLS-EU-Q47), and Brief Health Literacy Screen (BHLS). Some studies also used their own developed questionnaires. Table 3 shows some of the sources of health literacy measurement tools. All the tools used for measuring the health literacy of people with hearing impairment classify the level of health literacy into two to four degrees, and all have been tested for validity.

**Table 3:** Tools for measuring health literacy

First author	Tools	Evaluation methodology	Evaluation standards	Validity
Davis [43] (1993)	REALM	Read aloud the 66 medical terms and score points for the number of words pronounced correctly	Sum score (0–66) converted to 4 grades: 0–18: 3rd grade or less (Lower health literacy), 19–44: 4th through 6th grade (Lower health literacy), 45–60: 7th or 8th grade (Marginal health literacy), 61–66: 9th grade or more (Adequate health literacy)	SORT-R *r* = 0.96; PIAT-R *r* = 0.97; WRAT-R *r* = 0.88
Parker [44] (1995)	TOFHLA	Read and answer in a four-choice multiple-question format using materials used at the medical site to assess reading comprehension (50 questions) and math skills (17 questions)	Sum score (0–100) converted to 3 grades: 0–59: Inadequate health literacy, 60–74: Marginal health literacy, 75–100: Adequate health literacy	TOFHLA *r* = 0.91, REALM *r* = 0.80, Cronbach’s alpha = 0.98
Weiss [45] (2005)	NVS	Review the nutritional information tables in foods and assess whether you understand them correctly	Sum score (0–6) converted to 2 grades: 0–4: Limited health literacy, 4–6: Adequate health literacy	REALM *r* = 0.41, S-TOFHLA *r* = 0.61, Cronbach’s alpha = 0.76
McCormackL [46] (2010)	HLSI-SF	25 items consisting of multiple choice, T/F, and subjective questions, including the ability to read and understand documents, ability to find and interpret information (reading ability), ability to use quantitative information (repair ability), ability to listen effectively (oral ability), and ability to search for information via the Internet Assessment Method = Percent Correct (%)	Sum score (0–100) converted to 3 grades: ≥ 82: Proficient literacy 70–81: Basic literacy, < 70: Below basic literacy	S-TOFHLA *r* = 0.47, Cronbach’s alpha = 0.86
Sørensen [47] (2015)	HLS-EU-Q47	Answer 47 questions including obtaining, understanding, identifying, and using health information (very, 4 points—yes—difficult—very difficult, 1 point) Assessment method = (overall mean score—1) * (50/3)	Sum score converted to 4 grades: 0–25: Inadequate > 25–33: Problematic-limited > 33–42: Sufficient > 42–50: Excellent	Goodness-of-fit indices (0.9) in six Asian countries
Chew [48] (2004)	BHLS	The following two questions each have five level scores 1. How confident are you filling out medical forms by yourself? 2. How often do you have someone help you read hospital materials?	Sum score converted to 2 grades: < 9: Inadequate health literacy ≥ 9: Adequate health literacy	S-TOFHLA *r* = 0.42, REALM *r* = 0.40

As follows, we have summarized the findings of each article according to those listed in Table 4. Although each article does not show the same content due to differences in the purpose of the study, we can still summarize based on what is available.

**Table 4:** Current status of health literacy among people with hearing impairment and improvement methods

Study	Study aims	Main findings	Characteristics of health literacy problems	Difference in health literacy level within people with hearing disability	Barriers against health literacy	How to improve health literacy
**Gregg** [14]	To investigate the incorporation of Internet-based instruction regarding excellent health resources into the health curriculum of a deaf high school for hearing impaired pupils	A specific Internet health curriculum enhanced the ability of deaf high school pupils to find quality health information online	Students with hearing impairment often had difficulties in finding sources of health information	Not mentioned	Cultural sensitivity, discrepancies between ASL and English, disjointed patterns of interpersonal communication, and difficulty with medical terminology were all issues	Health science information courses helped students with hearing impairment access health information online
**Steinberg** [15]	To learn the details of the healthcare experiences of deaf people who communicate using American Sign Language	Communication issues were prevalent. Fear, mistrust, and frustration were the main problems. Many participants admitted to having little knowledge of their legal rights and thought that health care providers should learn more about the social components of deafness	People with hearing impairment had limited access to health information	Not mentioned	People with hearing impairment had communication barriers because translator services were insufficient, and writing was ineffectual due to syntax discrepancies between ASL and English	Improving communication between doctors and patients and addressing the cost of translation
**Groce** [17]	To determine if there were detectable differences in HIV/AIDS knowledge between hearing people and deaf sign language users in Swaziland	When compared to those with normal hearing, people with hearing disabilities were more likely (p0.05) to believe in inaccurate HIV transmission mechanisms and HIV prevention. 99% of hearing-impaired people had trouble speaking with healthcare workers	People with hearing impairment had knowledge gaps about HIV/AIDS and they have limited access to sources of health information	People with hearing loss had misconceptions about how HIV is transmitted, with 44% believing it can be transmitted through kissing; 42% trusted it can be transmitted through contact; 30% thought it can be transmitted through a dirty environment; and 52% said they do not know the route of mother-to-child transmission	Individuals with hearing disabilities had limited sources of health-related information and they had communication barriers	Disability-friendly sex education and materials need to be developed concerning the needs of people with hearing impairment
**Choe** [18]	To evaluate HIV awareness among Hard of Hearing and hearing people and determine how well HIV/AIDS messaging reaches deaf members of the community	There were differences in levels of comprehension (*p* < 0.05) regarding the aspects of how AIDS is propagated, and they found inequalities in information resources among the two groups	Cervical cancer was not well understood among people with hearing disabilities	Not mentioned	There was a need to deliver better cancer information and services to people with hearing loss in a way that they can understand	Cancer education activities are beneficial to persons who have hearing disabilities. By sharing relevant health information with others, participants can also contribute to the spread of knowledge
**Pollard** [19]	To see if seeing a graphically enhanced American Sign Language (ASL) cervical cancer education video could improve deaf women’s knowledge	Upon viewing the ASL cervical cancer education video, the treatment group gained more cancer knowledge than the control group who only received a basic education	Persons with hearing loss had a limited comprehension of medical terminology	68.4% of participants stated that they comprehended more than 90% of the health-related vocabulary, whereas 31.6% got scores that were deemed to reflect low health literacy	Reading and comprehension of English health-related terms and phrases were inadequate among individuals with hearing loss	Available health care education materials and programs should be developed to overcome knowledge gaps
**Hoang** [20]	To examine the health-related vocabulary knowledge of a group of deaf adults	On the modified REALM task, 32% of deaf adults received results comparable to REALM levels considered indicative of low health literacy. The patterns of least and most used words diverge from what auditory REALM respondents expect	People with hearing impairment had low health literacy due to language barriers, and health care providers reported a limited understanding of deaf culture	Not mentioned	Health service providers lacked training in deaf culture and there were a limited number of medical staff who could speak sign language	To improve clinicians’ cultural competency through the Deaf Community Training program (DCT), exposure to members of the deaf community in a clinical setting, the classes, or self-paced learning modules
**Convery** [21]	To evaluate the role of the Deaf Community Training (DCT) program on medical students’ understanding of deaf culture and compare it with that of teachers	DCT medical students had a better understanding of deaf culture and patients with hearing impairment than non-DCT medical students and instructors	Some people with hearing impairment have difficulty understanding the text in the instructions and the accompanying pictures	Not mentioned	Not mentioned	Larger fonts, graphical representations, and non-specialist jargon should be utilized to increase information accessibility for a broader variety of hearing aid users
**Maddalena** [22]	To ascertain the proportion of hearing-impaired people who can effectively select and construct a hearing aid using illustrated written instructions, and who can place the device into their ear with or without the assistance of a partner	Participants’ ability to complete the assembly activity independently and accurately was greatly influenced by their level of health literacy. Higher levels of health literacy were connected with a greater likelihood of completing tasks independently and successfully	People with hearing impairment had inadequate experience with death and a poor understanding of accessible palliative care services	Not mentioned	Persons with hearing loss had communication challenges with doctors, while healthcare providers had minimal knowledge of deaf culture	ASL interpreters were required to assist with communication in healthcare facilities, and because deaf people had varying levels of ASL fluency, personalized communication is required
**Yao** [23]	To investigate and explain Deaf people’s and their carers’ experiences at the end of a patient’s life, and to investigate the barriers that impact Deaf people’s choice to the health care system utilization during the terminal condition	Participants had little experience with dying, little understanding of accessible palliative care resources, and healthcare workers had inadequate knowledge of deaf culture. There are also some communication issues between deaf people and health professionals	In the cancer knowledge categories, women with hearing loss had poorer baseline knowledge than hearing individuals	94.5% of people with hearing loss reported “very easy to somewhat easy” to receive information from the video	Communication difficulties restricted people with hearing loss from getting health information and services	The educational video on cervical cancer can provide knowledge about cancer for the hearing impaired
**Berman** [24]	To compare the change in the cancer knowledge in deaf women and hearing women before and after watching a vividly enhanced educational video about cervical cancer	Hearing women performed better before watching the video. After the intervention, both groups showed a substantial increase in general and cervical cancer knowledge	People with hearing disabilities had misunderstandings about breast cancer risk factors, screening, and management	Just 64.2% of participants successfully classified the purpose of mammography	In clinical settings, people with hearing impairment encountered considerable challenges in getting information, referrals, and assistance	Breast cancer education programs, insurance providers advising hearing loss women of optimal health care practices, and health care providers promoting awareness about people with hearing impairment can all increase health literacy
**Jensen** [25]	To report the baseline characteristics of the D/deaf women and to test the program they designed to be available to D/deaf women with different education levels	Breast cancer risk factors, screening, and therapy are all misunderstood by the deaf. 64.2% of those surveyed correctly identified the aim of mammograms	Women with hearing disabilities had poorer cancer knowledge ratings than hearing women before the intervention	64% of women with hearing loss said the video was “very easy,” 28% thought it was”somewhat easy” to understand, 6% thought it was “somewhat difficult,” and 2% thought it was “very difficult” to understand	Language and cultural barriers to health information and care were mentioned by people with hearing impairment	A video-based method to educate women with hearing disabilities is practically meaningful
**Convery** [26]	To see if an ovarian cancer education video in American Sign Language with English annotation and voice-over could help deaf and hearing women bridge the knowledge gap	Hearing women scored considerably higher on the pre-test than deaf women. All the deaf women’s knowledge scores had grown after the intervention, and hearing women’s knowledge had also increased, which lead to a new gap	Some people with hearing impairment have difficulty understanding the guidance documents and are unable to fit hearing aids independently	Not mentioned	Not mentioned	The help of a partner may improve the health literacy of people with hearing impairment
**Mckee** [27]	To assess hearing-impaired adults and their partners’ ability to operate a pair of BTE hearing aids by a set of written and visual instructions	Better health literacy was substantially related to independent task completion in both two country individuals. Task accuracy was substantially related to greater levels of cognitive functioning among South African participants	Hearing impaired people struggle to obtain health information through the media, and health care communication	In the Peabody Individual Achievement Test-Revised, 74% of the hearing impaired had a reading level below grade 8, 26% were equal to or above grade 8, and the average reading level of the participants was 5.9	Communication and linguistic obstacles, as well as social marginalization, low education, and low income, are all present	Sign language videos can be effective in helping people with hearing impairment access and understand health information
**Ferguson** [28]	To assess the prevalence and relationship of limited health literacy among deaf ASL users and hearing people	48% of deaf people had low health literacy, and deaf participants were 6.9 times more likely than hearing individuals to have limited health literacy	People with hearing impairment lacked an understanding of pharmaceutical instructions and services in pharmacies	Not mentioned	There were difficulties in communication between people with hearing impairment and pharmacy workers, and there was also a need for more understanding and tolerance between them	Pharmacists need to become more culturally sensitive and competent in their interactions with people with hearing loss and this can be achieved through education and training
**Smith** [29]	To explore communication challenges and needs for deaf and hard-of-hearing (HOH) patients when they find medical care	Deaf and HOH patients have special needs that pharmacists need to comprehend and solve. Proper communication and literacy evaluation is a key to guarantee safe medication use and first-rank health outcomes	People with hearing loss had inadequate knowledge about cardiovascular disease and have trouble getting health information	Adolescents with hearing impairment had inconsistent knowledge of vascular health, with many unable to identify the reasons or start of heart attacks and strokes and just a few able to appropriately characterize cholesterol	Communication barriers with health care providers, difficulty to understand the jargon in printed health information, and ineffective school health education programs were all problems	Improve interpreter education and disseminate information through social media and create accessible and culturally appropriate health surveys and health education initiatives
**Kushalnagar** [30]	To eventually use the knowledge gathered to enhance the delivery of cardiovascular health education	Family, health education instructors, healthcare practitioners, written materials, and informal sources were selected as the top five sources of cardiovascular health information	People with hearing impairment with poorer health literacy had a harder time finding information on ASL-accessible health websites	Subjects who indicated ASL as their main language had worse health literacy scores than those who said they favored both languages equally. Furthermore, primary ASL speakers showed poorer levels of health literacy	Whether ASL’s health website is straightforward to use, browse, and comprehend	The health website should not only be available in ASL, but it should also be easy to use. Furthermore, short, clear ASL videos can encourage persons with hearing loss to seek medical attention and communicate any difficulties with their physicians
**Smith** [31]	To learn about ASL users’ experiences navigating these websites, and their minds on the understanding of the contents	Participants mentioned the benefits of adding captioning and a signer model to health videos. Subjects who had limited health literacy had greater difficulty finding information on the website	Even when functional health knowledge was controlled for, there were still disparities in general health knowledge and cardiovascular health knowledge measures between hearing-impaired and hearing adolescents	Hearing-impaired adolescents with higher health literacy reported having better hearing assistive devices and using them regularly, describing English as their best language, having excellent communication with their parents, and attending hearing schools at least half of the time	Cultural, social, and familial communication issues may all have an impact on the formation of healthy knowledge in teenagers with hearing loss	Improving hearing loss adolescent health literacy should focus on health-related dialogues with their families, access to written health information, and access to relevant information from health care practitioners and educators
**Haricharan** [32]	Quantifying health literacy in students with hearing impairment and auditory adolescents	People with hearing impairment have inadequate health literacy, which can be improved by intervening in their health-related talks with their family	Although the intervention increased participants’ general awareness of hypertension and healthy living, they still found the medical terminology challenging to understand	SMSs were easy to interpret for 78% of participants but difficult for 13%	People with hearing disabilities had little exposure to health information	Text messaging can enhance the health literacy of people with hearing loss, but it needs to take into account the unique needs and communication preferences of people with hearing disabilities
**Palmer** [33]	To assess the availability of using short message service (SMSs) in deaf people	An improvement in hypertension knowledge and healthy living in participants; 6 in 19 questions’ answers also showed a significant change, but the medical terminology is still hard to understand. Sone ways to enhance SMS campaigns were identified	People with hearing disabilities had difficulty accessing and understanding health information effectively	Hearing impaired participants with a high level of education showed a significant increase in health knowledge whether they were taught bilingually or monolingually, but participants with a low level of education could increase their knowledge only when they were taught bilingually	Differences in the primary language and educational levels of people with hearing disabilities can be a source of difficulty in accessing health information	Online non-face-to-face teaching using a bilingual approach can enable people with hearing impairment to access health information effectively
**Kushalnagar** [34]	To investigate the use of bilingual (ASL with English closed captioning) versus monolingual (English text) online cancer genomics information	There was a significant interaction between linguistic modality, education, and change in knowledge scores (*p* = .01). Regardless of modality, the high education group increased knowledge (Bilingual: p.001; d = .56; Monolingual: p.001; d = 1.08) while bilingual (p.001; d = .85) but not monolingual (*p* = .79; d = .08) modality boosted knowledge in the low education group	Because they were unable to hear health-related information on television, radio, or public broadcasting, many hearing-impaired people are not fully exposed to health information and must rely on family members or peers for information	Not mentioned	Due to communication problems in the home or health environment, people with hearing disabilities had fewer opportunities to obtain health information	Sign-language-enabled social media can enhance health knowledge and self-advocacy health care practices through interactive health literacy exercises
**Pinilla** [35]	To explore the association between critical health literacy (CHL) and discussion of health information among participants	The discussion with friends about health-related information is associated with CHL in two groups, while the discussion with family was only related to hearing people	Participants did not actively seek information about diabetes unless they were surrounded by others with the disease or had it themselves	27% of participants knew of two different types of diabetes, 67% had only heard of the disease but were unsure what it was, and one respondent had never heard of diabetes	All participants reported communication challenges as a result of health care providers’ lack of sign language expertise and a scarcity of interpreters	Improve communication between healthcare professionals and patients by incorporating content on common sign language communication skills into medical communication classes or training courses for health service staff
**Stevens** [36]	To investigate illness ideas encoded in signs, main noncommunicable disease preventive behavior, and communication hurdles among deaf community members	Personal spoken and written language literacy influences health information-seeking behavior among deaf people. Breaking down the walls is critical for establishing a better understanding of diabetes and other disease preventive programs	People with hearing impairment had difficulty receiving and comprehending health information in a healthcare setting	People over the age of 85 years were more likely than the younger group to report misinterpreting medical information conveyed by nurses and doctors	Every interaction between health care providers and people with hearing impairment had inadequate communication and errors	To improve one-on-one provider interactions, avoid phone calls, and provide health information in writing
**Gur** [37]	To evaluate the experiences of people with hearing impairment in the healthcare setting to identify confusion and unsatisfied needs and provide feasibility recommendations	Three communication circumstances stood out as frequently generating communication challenges between patients and providers. Although 93% of participants said that they informed providers about their hearing impairment, 29.3% of all participants claimed that no adjustments were taken	Had trouble connecting with healthcare providers, made mistakes when taking doctor-prescribed medication, could not grasp prescription drug instructions, and did not understand documents and texts provided by the institution	70.5% of individuals with hearing loss had insufficient health literacy, 19.3% had restricted health literacy, 2.3% had acceptable health literacy, and 8% had exceptional health literacy	People with hearing loss had difficulty communicating with their doctors and taking their medications	Medication instructions can be provided in sign language or writing by health care professionals, and the hearing impaired and health care providers treat each other with respect and improve their relationship
**Tolisano** [38]	To establish the levels of health literacy among D/HoH teenagers, as well as the challenges and misunderstandings they face while applying for healthcare services	Students who had difficulty communicating with their doctor, administering medication, etc. had low health literacy and wanted their doctor to be able to communicate in sign language or writing. Hearing impairment and health literacy were shown to have a strong association. (*R* = 0.659, R2 = 0.434) (*p* < 0.01)	People with hearing impairment had difficulty reading text and accessing health information	Not mentioned	Hearing impaired people had language and reading difficulties	To develop a tool to help people with hearing impairment understand health information
**Wells** [39]	To better understand the impact of hearing impairment on patient health literacy	9.7% of patients were found to have inadequate health literacy. 284 (95%) patients had available hearing data, of whom 235 (82.7%) had Grade A or B hearing and 49 (17.3%) had Grade C or D hearing. Patients with Grade C or D hearing had a lower median brief health literacy screen (BHLS) composite score (11.6 versus 13.6, *p* < 0.0001) and an increased rate of inadequate health knowledge (28.6% versus 4.7%, OR = 8.15, 95% CI 3.42–19.37) compared to those with Grade A or B hearing	Health literacy was classified based on one’s confidence in completing medical forms, with responses ranging from a little to not at all labeled as LHL and extreme, quite a little, and somewhat classified as sufficient health knowledge	Individuals with unassisted severe hearing loss were 80% more likely to report limited health literacy (LHL), followed by those with unaided mild hearing loss (46%), aided severe hearing loss (41%), and aided mild hearing loss (4%); those with aided moderate hearing loss were not at elevated risk for LHL	Low health literacy is associated with older age, male gender, poorer income, hearing loss, and not utilizing hearing aids	Hearing aids could be used to increase health literacy
**Willink** [40]	To investigate the factors related with low health literacy (LHL), health care costs, and treatment gaps based on health literacy, hearing impairment, and cochlear usage status	7% had LHL, whereas 41% suffered hearing loss. Hearing loss, particularly severe unassisted hearing loss, was linked to LHL. LHL will result in greater annual medical costs than individuals with good health literacy, whether they have hearing loss	People with hearing impairment had difficulties in understanding health insurance	Among those who had a lot of difficulty hearing, 21% said they had a little issue, and 28% said they had a lot of trouble locating Medicare information because of their hearing. Those who had a little difficulty hearing reported they had a little (12%) issue and a lot of (3%) trouble locating Medicare information because of their hearing	The methods available to increase Medicare knowledge were not created for persons with hearing problems and did not employ language that people with hearing loss are comfortable with. Furthermore, the system provided limited and variable coverage for hearing loss	More outreach to those who have hearing loss to generate relevant materials and ensure that enough resources are accessible to orient and comprehend the system
**Almusawi** [41]	To investigate how hearing loss affects Medicare beneficiaries’ comprehension of the Medicare program, their ability to make comparisons and analyses of health plans, and their comfort level with the information provided	Medicare beneficiaries with a little or a lot of trouble hearing had 18% and 25%, increased odds, respectively, of reporting difficulty with understanding Medicare, compared with those with no hearing trouble. About one in five Medicare beneficiaries with hearing loss identified that their hearing made it difficult to find Medicare information	People with hearing impairment have communication barriers and limited sources of health information	Only 30% of persons with hearing impairment were aware of the availability of medical counseling services during isolation, whereas 15% were aware of written therapy services	People with different dialects and sign languages cannot interact with one another, and health information sources are restricted	Increase sign language training and assistance to develop accessible and understanding medical information for individuals with hearing impairment
**Tran** [42]	To evaluate whether health literacy is related to the degree of hearing loss at the time of the initial hearing examination and the adoption of hearing aids by hearing aid patients	Patients with LHL had a higher likelihood of having significant hearing loss (adjusted mean pure-tone average [PTA] difference, 5.38 dB, 95% confidence interval [CI] 2.75–8.01). Health literacy was not connected to the cochlear adoption rate among hearing aid patients (odds ratio [OR] 0.85, 95% CI 0.40–1.76)	Patients with LHL had a higher likelihood of having significant hearing loss	At the time of first testing, low health literacy was linked to more severe hearing loss	People with hearing loss were difficult to enter the hearing healthcare system or they were not confident to do this	Not mentioned
	To evaluate the discrepancies in COVID-19 health knowledge and practice between two groups	Hearing loss and using sign language as the major way of communication were both linked to reduced health literacy. Some differences in the utilization of health information sources				

### Health literacy levels of people with hearing impairment

The prevalence of inadequate health literacy among people with hearing impairment is high. Mckee et al. [27] conducted a study with 166 participants with hearing loss and 239 hearing participants without, and showed that 48% of participants with hearing loss have inadequate health literacy and were 6.9 times more likely than hearing people to have poor health literacy. Pollard et al. [19] investigated health-related vocabulary knowledge in a sample of adults through a modified REALM and found that 32% of people with hearing loss received scores comparable to low health literacy scores; all of their study participants were people with hearing disabilities and 80.8% obtained a college degree, implying that even people with hearing disabilities with high or average levels of education may be at risk of lower health literacy. Tolisano et al. [38] found ratings of participants’ hearing were categorized into four grades, and patients with poorer hearing grades C and D had lower health literacy scores measured with the BHLS than those with grades A and B (BHLS score in the range of 11.6–13.6). Wells et al. [39] divided 19,223 older adults into five groups based on their self-reported hearing disability level, including those with severe unaided hearing loss, severe with hearing aid use, mild with assistance, mild without assistance, and no hearing loss. Of these, the unaided mild, aided severe and unaided severe hearing loss groups showed lower health literacy than the other groups, although this connection diminished with the use of hearing aids.

### The barriers to health literacy for people with hearing impairment

Among the people with hearing impairment, there is a lack of knowledge and misconceptions about diseases, such as cervical cancer [18, 23], ovarian cancer [25], breast cancer [24], HIV/AIDS [17], COVID-19 [41], and diabetes [35]. People with hearing impairment also had limited access to health information and encounter difficulties in seeking health information [49]. Several studies were conducted on a hearing-impaired group and a control group to compare their knowledge on cervical, ovarian, and breast cancers after listening to a graphic-rich video in American Sign Language with English subtitles regarding cancer [18, 23, 25]. The studies obtained similar results; women in the control group performed better before watching the video, while following the intervention, both groups’ knowledge on relevant tumors improved significantly [18, 23–25]. These findings imply that the health literacy of people with hearing impairment can be improved if there are more effective ways to expose them to health information and provide more access to it.

People with hearing impairment tend to visit their doctors more often than those with good hearing, but they can have more difficulty communicating with professionals [50], which is consistent with the findings of a survey of American Sign Language (ASL) interpreters [51]. In Stevens et al.’s study [36], more than 90% of people with hearing impairment reported problems with poorly communicated information and communication difficulties when their name was called with the presenter’s back being turned, as well as communication over the telephone, which can affect the quality of patient care, satisfaction, and health outcomes. According to Steinberg et al. [15], adults with hearing impairment who used ASL were distrustful, fearful, and frustrated with medical care and believed that if doctors could communicate with them in sign language or with a live interpreter, it would help them establish good communication with the doctor and improve their satisfaction with the medical service. Similar communication challenges were observed among teenagers with hearing disabilities [37], with 55.7% preferring written prescriptions or care procedures and 87.5% preferring sign language communication. A study [28] has shown that when pharmacists lack patience or understanding of the real needs of people with hearing impairment when they seek medication care, the bond between them can be weakened and, in turn, can affect the safe administration of medication to patients. All of these findings illustrate the importance of effective communication between patients and doctors about medication, treatment, and health outcomes.

### The impact of health literacy levels on health management among people with hearing impairment

People with both hearing impairment and limited health literacy were more likely to have higher medical costs [39]. In addition, people with hearing loss have very limited awareness of health insurance and have little access to the related information [40]. Willink et al. [40] discovered that Medicare participants who had a little or a lot of hearing impairment were 18% and 25%, respectively, more likely to report having difficulty understanding Medicare than those who did not have hearing problems. Approximately 20% of Medicare enrollees with hearing loss reported that their hearing disability made it hard to find information about Medicare. This also implies that methods to assist people with hearing loss in understanding Medicare should be consistently developed.

### Methods for improving health literacy

Online education can help enhance the health knowledge of people with hearing impairment as well as their capacity to seek health information on the internet [14, 18, 23, 25, 33]. Palmer et al. [33] reported that the bilingual modality via both the signer and closed captioning provided better access to cancer genetics information for less-educated ASL users compared to the monolingual modality. This study supports that materials prepared with sign language and extra captioning and graphics improve the sign language user’s understanding and satisfaction. Short Message Service (SMS) can help people with hearing loss become more aware of hypertension and healthy living. However, because of the special demands and preferences of sign language users, SMS services must be investigated further to fulfill the needs of the hearing impaired. Haricharan et al. [32] suggested numerous strategies to improve SMS campaigns for people with hearing disabilities, including the use of images, combination of SMS with signed drama, use of 'signed’ SMS, and linkage of SMS campaigns to interactive communication services.

Additionally, communication issues between people with hearing impairment and health care providers need to be addressed. Most people that are hard-of-hearing in the survey mentioned difficulties in communicating with doctors, the obscurity of some terminology, and even misunderstandings [36]. They hoped to communicate with the doctor in sign language or have a sign language interpreter on-site to help them speak with their doctor, which would increase their satisfaction with health services, strengthen the doctor-patient relationship, and improve their health outcomes [15, 36, 37]. To facilitate communication between patients with hearing impairments and healthcare professionals, it is essential to pre-educate healthcare professionals about people with hearing loss [20]. In Hoang et al.’s study, medical students were divided into two groups based on whether they had undertaken a community education program on hearing impairment. When knowledge of the overall “deaf culture “ in the healthcare setting was assessed, the summary scores varied widely—26.9 for those who had completed the education program, 17.1 for the medical school faculty, and 13.8 for those who had not completed the education program [20]. Overall, the faculty members scored similarly to medical students who did not complete the educational program on issues particularly relevant to interactions with hearing loss patients. This research proved that primary education is crucial in interacting with deaf patients, regardless of clinical expertise [20].

## Discussion

This study assessed the existing literature relating to the health literacy of people with hearing impairment. There are some common observations showing that people with hearing impairment have low health literacy and have difficulties communicating with health providers and understanding—and often misunderstanding—when seeking medical services [15]. They also have limited sources of relevant health information, incomplete knowledge of diseases, health insurance, and even misunderstandings of the same [35, 40, 41, 49]. In addition, participants were under-informed about the palliative care services available to them, and health providers were unknown about the deaf culture [22].

Of our selected literature, 25 were evaluated as having a “low” risk of bias because most studies used instruments to measure health literacy or conducted case–control group studies. Two were deemed to be at high risk of bias, owing to some confounding variables and missing data in the research, which influenced the results. The other two articles were judged to be having an “unclear” risk of bias due to the complete inconsistency of the three main domains. This showed that for the people with hearing impairment, high-quality research has been conducted as enough comprehensive factors were considered in the study to minimize possible bias.

Few studies have investigated health information sources for people with hearing impairment. Smith et al. (2015) interviewed hearing loss adolescents who used sign language and identified five main sources of cardiovascular health information, including family, health education teachers, health care providers, print, and informal sources. They suggested that communal funds be used to conduct appropriate health surveys and identify health education programs, enhance interpreter education, and distribute information using social media. All three authors of this literature [29] are also people with hearing disabilities; thus, they researched a group to which they belong and understand, which is one way that future research on people with disabilities could be conducted—by researchers who know enough about particular groups.

Increased health literacy through conversational means is crucial, and because of the influence of independence from their families, college students who lose their hearing often rely on friends to get health information and education [34]. Mutual sign language education with friends, discussion, and participation in these early social discussions can improve overall health knowledge. Recent improvements in online information technology, social networks that enable the gathering of sign language users, and frequent gatherings through online virtual meetings can provide them a space for dialogues, potentially sharing their most recent health information [34]. Another option is to employ SMS functionality, which in 2015 had a nationwide penetration rate of 85.67% in South Korea; this value will reach 97.4% by 2025 [52]. We also anticipate that text messaging will become increasingly crucial as more cellphone apps and technology are developed for individuals with disabilities [53, 54]. Haricharan et al. [32] previously investigated sending regular text messages to boost health information and hypertension awareness among people with impairments. The content contained 20 well-designed hypertension messages, 57 food habits, methods to avoid hypertension through healthy living—such as exercise—and four activities [32]. Although some literature recommend the use of written materials [31, 36, 37], we must recognize that people with hearing impairment who speak sign language as their first language frequently struggle to interpret written texts and lack literacy in this area [15, 55]. This is especially true for people who are more likely to have a low level of education [33]. In Korea, the hearing handicapped commonly employ Korean Sign Language, lip-synthesis, and notes, among other means [55]. According to the 2017 Korean Sign Language Use Survey, 69.3% of people with hearing loss use sign language as their primary form of communication, but only 30.5% fully understand it [56]. Hence, using textual material to spread health messages in Korea may be a good option.

In summary, our literature review indicate several currently feasible ways to improve the health literacy of people with hearing impairments: health literacy education for people with hearing impairments, hearing disability-related education for healthcare professionals to help understand better the needs of people with hearing impairments, and popularization of the use of smartphones (e.g., SMS). In addition, if we can analyze subgroups of people with hearing impairment by conditions such as whether they use hearing aids or not and whether they know sign language or not in subsequent studies, that would help to target people with hearing impairment to improve their health literacy.

There are several limitations to this study. First, it is hard to generalize the selected literature as there were variations by countries in selection criteria for people with hearing impairments, age distribution and number of participants, and methods and tools used. Second, it was not possible to compare the selected foreign studies with the Korean results as there are no national studies on health literacy among people with hearing impairment. Third, although the literature we collected was published over a wide time period, from 2000 to 2021, there are not many studies on health literacy among people with hearing impairment. Each paper defined low health literacy differently and, to our knowledge, there is not a clear standard at present. Paul et al. [57] summarized 43 different health literacy instruments and illustrated that the quality of these instruments varied considerably according to their psychometric properties. Regardless, overall, the literature is of high quality and contains information on the low health literacy of people with hearing impairment, dilemmas faced in the healthcare setting, limited sources of medical information, and some feasible ways to improve it. These existing relevant articles can inform about the current state of health literacy among people with hearing loss and ways of improving the health literacy of people with hearing impairment.

## Conclusions

People with hearing impairment have shown a willingness to seek out and learn about health information. However, because of the complex terminology, a lack of easily available health information resources and illustrations by handicap type has been discovered. The recent continuous development of technology for people with disabilities and easy access to smartphones have correspondingly provided more and varied options for people with disabilities to receive health information. This suggests that developing new technology and policies for people with hearing impairment is necessary not to mention promoting provision of information via sign language. For instance, enforcement policies would be required for the existing app technologies in the uploading of health information for various chronic diseases, such that deafness characteristics are taken into consideration and people subscribe only to the information they require.

### Supplementary Information

**Additional file 1.** Described the specific literature search methods in the PubMed, Cochrane and Embase databases.

**Additional file 2.** Demonstrated the key questions that we came to select from the corresponding literature.

**Additional file 3.** Described the result of the assessment of research quality using RoBANs.

## Data Availability

Not applicable.

## Abbreviations

IOM: Institute of Medicine
PRISMA: Preferred Reporting Items for Systematic Reviews and Meta-analysis
MeSH: Medical Subject Headings
PICOTS-SD: Participants, Interventions, Comparisons, Outcomes, Timing, Settings, Study Design
PICO: Participants, Intervention, Comparison, Outcome
RoBANS: Risk of Bias Assessment Tool for Nonrandomized Studies
REALM: Rapid Estimate of Adult Literacy in Medicine
TOFHLA: Test of Functional Health Literacy in Adults
NVS: Newest Vital Sign
HLSI: Health Literacy Skills Instrument
HLS-EU-Q47: European Health Literacy Survey
BHLS: Brief Health Literacy Screen
ASL: American Sign Language
SMS: Short Message Service
