# Supplementary material for: Exploring the health literacy status of people with hearing impairment: a systematic review
Source: Arch Public Health. 2023 Nov 22;81:206. doi: 10.1186/s13690-023-01216-x (PMC10664265; doi:10.1186/s13690-023-01216-x)
Supplement: Supplementary file 1 — Additional file 1. Described the specific literature search methods in the PubMed, Cochrane and Embase databases. [file 13690_2023_1216_MOESM1_ESM.docx]

**Additional file 1. Literature search methods in difference databases**

| Database | Search Method |
| --- | --- |
| PubMed | (hearing loss or persons with hearing impairments or deafness[MeSH Terms]) AND (health literacy) AND (2000:2021[pdat]) Filters: from 2000 - 2021 |
| Embase | 'hearing impairment'/exp AND 'health literacy' AND [2000-2021] |
| Cochrane | #1 hearing loss with Publication Year from 2000 to 2021, in Trials  #2 health literacy with Publication Year from 2000 to 2021, in Trials  #3 #1 and #2 with Publication Year from 2000 to 2021, in Trials |
