# Supplementary material for: Exploring the health literacy status of people with hearing impairment: a systematic review
Source: Arch Public Health. 2023 Nov 22;81:206. doi: 10.1186/s13690-023-01216-x (PMC10664265; doi:10.1186/s13690-023-01216-x)
Supplement: Supplementary file 2 — Additional file 2. Demonstrated the key questions that we came to select from the corresponding literature. [file 13690_2023_1216_MOESM2_ESM.docx]

**Additional file 2. Key questions in the study PICOTS-SD (Participants, Intervention, Comparison, Outcome, Timing, Setting, Study Design)**

| **Participants(P)** | People with hearing impairment. |
| --- | --- |
| **Intervention(I)** | The definition of health literacy   - Health literacy is about knowing what affects your health, the health of your family, and the health of your community, and how to deal with these influences. - Patient’s ability to read and understand prescribed medications, appointment slips， and other basic health materials. - Defined as a cognitive and social skill that determines an individual’s motivation, ability to access information, and understand and use that information to promote and maintain health. - Gain the knowledge, skills, and confidence to improve the health of individuals and communities through changes in their lifestyles and living conditions. |
| **Comparison(C)** | Non-disabled or without impairments. |
| **Outcome(O)** | Level of health literacy, Influencing factors, Measurement methods, Health management. |
| **Timing** | Research for the period 2000–2021. |
| **Setting** | No setting restrictions. |
| **Study Design** | Non-randomized studies, observational studies, structured interviews, semi-structured interviews, quantitative or qualitative analysis studies. |
