# Supplementary material for: Exploring the health literacy status of people with hearing impairment: a systematic review
Source: Arch Public Health. 2023 Nov 22;81:206. doi: 10.1186/s13690-023-01216-x (PMC10664265; doi:10.1186/s13690-023-01216-x)
Supplement: Supplementary file 3 — Additional file 3. Described the result of the assessment of research quality using RoBANs. [file 13690_2023_1216_MOESM3_ESM.docx]

**Additional file 3. Research bias risk assessment form using RoBANS**

| **Domains** | The selection of participants | Confounding variables | The measurement of exposure | The blinding of the outcome assessments | Incomplete outcome data | Selective outcome reporting |
| --- | --- | --- | --- | --- | --- | --- |
| **Instructions**  **First author**  **(year)** | Bias of choice due to inadequate selection of objects | Selection bias due to improper checking and consideration of confounding variables | Execution bias due to inappropriate intervention (exposure) measurements | Bias in the determination of results due to the blinding of the outcome assessments | Bias due to improper handling of incomplete outcome data | Bias due to selective outcome reporting |
| Gregg (2002)^33^ | ■ Low  □ High  □ Unclear | □ Low  ■ High  □ Unclear | ■ Low  □ High  □ Unclear | □ Low  □ High  ■ Unclear | □ Low  ■ High  □ Unclear | ■ Low  □ High  □ Unclear |
| Steinberg (2006)^29^ | ■ Low  □ High  □ Unclear | ■ Low  □ High  □ Unclear | □ Low  ■ High  □ Unclear | □ Low  □ High  ■ Unclear | ■ Low  □ High  □ Unclear | ■ Low  □ High  □ Unclear |
| Groce (2006)^48^ | ■ Low  □ High  □ Unclear | □ Low  ■ High  □ Unclear | ■ Low  □ High  □ Unclear | ■ Low  □ High  □ Unclear | ■ Low  □ High  □ Unclear | ■ Low  □ High  □ Unclear |
| Groce (2007)^22^ | ■ Low  □ High  □ Unclear | ■ Low  □ High  □ Unclear | ■ Low  □ High  □ Unclear | ■ Low  □ High  □ Unclear | ■ Low  □ High  □ Unclear | ■ Low  □ High  □ Unclear |
| Choe (2009)^18^ | ■ Low  □ High  □ Unclear | ■ Low  □ High  □ Unclear | ■ Low  □ High  □ Unclear | ■ Low  □ High  □ Unclear | ■ Low  □ High  □ Unclear | ■ Low  □ High  □ Unclear |
| Pollard (2009)^15^ | ■ Low  □ High  □ Unclear | □ Low  ■ High  □ Unclear | ■ Low  □ High  □ Unclear | ■ Low  □ High  □ Unclear | ■ Low  □ High  □ Unclear | ■ Low  □ High  □ Unclear |
| Hoang (2011)^36^ | ■ Low  □ High  □ Unclear | ■ Low  □ High  □ Unclear | ■ Low  □ High  □ Unclear | □ Low  □ High  ■ Unclear | ■ Low  □ High  □ Unclear | ■ Low  □ High  □ Unclear |
| Convery (2011)^49^ | ■ Low  □ High  □ Unclear | ■ Low  □ High  □ Unclear | ■ Low  □ High  □ Unclear | ■ Low  □ High  □ Unclear | ■ Low  □ High  □ Unclear | ■ Low  □ High  □ Unclear |
| Maddalena (2012)^37^ | □ Low  ■ High  □ Unclear | □ Low  □ High  ■ Unclear | □ Low  ■ High  □ Unclear | □ Low  □ High  ■ Unclear | ■ Low  □ High  □ Unclear | ■ Low  □ High  □ Unclear |
| Yao (2012)^19^ | ■ Low  □ High  □ Unclear | ■ Low  □ High  □ Unclear | ■ Low  □ High  □ Unclear | ■ Low  □ High  □ Unclear | ■ Low  □ High  □ Unclear | ■ Low  □ High  □ Unclear |
| Berman (2013)^21^ | ■ Low  □ High  □ Unclear | ■ Low  □ High  □ Unclear | □ Low  ■ High  □ Unclear | ■ Low  □ High  □ Unclear | ■ Low  □ High  □ Unclear | ■ Low  □ High  □ Unclear |
| Jensen (2013)^20^ | ■ Low  □ High  □ Unclear | ■ Low  □ High  □ Unclear | ■ Low  □ High  □ Unclear | ■ Low  □ High  □ Unclear | ■ Low  □ High  □ Unclear | ■ Low  □ High  □ Unclear |
| Convery (2013)^50^ | ■ Low  □ High  □ Unclear | ■ Low  □ High  □ Unclear | ■ Low  □ High  □ Unclear | ■ Low  □ High  □ Unclear | ■ Low  □ High  □ Unclear | ■ Low  □ High  □ Unclear |
| Mckee (2015)^14^ | ■ Low  □ High  □ Unclear | ■ Low  □ High  □ Unclear | ■ Low  □ High  □ Unclear | ■ Low  □ High  □ Unclear | ■ Low  □ High  □ Unclear | ■ Low  □ High  □ Unclear |
| Ferguson (2015)^31^ | ■ Low  □ High  □ Unclear | ■ Low  □ High  □ Unclear | □ Low  ■ High  □ Unclear | □ Low  □ High  ■ Unclear | ■ Low  □ High  □ Unclear | ■ Low  □ High  □ Unclear |
| Smith (2015)^38^ | ■ Low  □ High  □ Unclear | □ Low  ■ High  □ Unclear | □ Low  ■ High  □ Unclear | □ Low  □ High  ■ Unclear | ■ Low  □ High  □ Unclear | ■ Low  □ High  □ Unclear |
| Kushalnagar (2015)^51^ | ■ Low  □ High  □ Unclear | ■ Low  □ High  □ Unclear | ■ Low  □ High  □ Unclear | ■ Low  □ High  □ Unclear | ■ Low  □ High  □ Unclear | ■ Low  □ High  □ Unclear |
| Smith (2016)^43^ | ■ Low  □ High  □ Unclear | ■ Low  □ High  □ Unclear | ■ Low  □ High  □ Unclear | ■ Low  □ High  □ Unclear | ■ Low  □ High  □ Unclear | ■ Low  □ High  □ Unclear |
| Haricharan (2017)^35^ | ■ Low  □ High  □ Unclear | ■ Low  □ High  □ Unclear | ■ Low  □ High  □ Unclear | ■ Low  □ High  □ Unclear | □ Low  ■ High  □ Unclear | ■ Low  □ High  □ Unclear |
| Palmer (2017)^34^ | ■ Low  □ High  □ Unclear | ■ Low  □ High  □ Unclear | ■ Low  □ High  □ Unclear | ■ Low  □ High  □ Unclear | ■ Low  □ High  □ Unclear | ■ Low  □ High  □ Unclear |
| Kushalnagar (2017)^39^ | ■ Low  □ High  □ Unclear | □ Low  ■ High  □ Unclear | ■ Low  □ High  □ Unclear | ■ Low  □ High  □ Unclear | ■ Low  □ High  □ Unclear | ■ Low  □ High  □ Unclear |
| Pinilla (2019)^24^ | □ Low  ■ High  □ Unclear | □ Low  □ High  ■ Unclear | □ Low  ■ High  □ Unclear | □ Low  □ High  ■ Unclear | ■ Low  □ High  □ Unclear | ■ Low  □ High  □ Unclear |
| Stevens (2019)^28^ | ■ Low  □ High  □ Unclear | □ Low  ■ High  □ Unclear | ■ Low  □ High  □ Unclear | ■ Low  □ High  □ Unclear | ■ Low  □ High  □ Unclear | ■ Low  □ High  □ Unclear |
| Gur (2020)^30^ | ■ Low  □ High  □ Unclear | ■ Low  □ High  □ Unclear | ■ Low  □ High  □ Unclear | ■ Low  □ High  □ Unclear | ■ Low  □ High  □ Unclear | ■ Low  □ High  □ Unclear |
| Tolisano (2020)^16^ | ■ Low  □ High  □ Unclear | □ Low  ■ High  □ Unclear | ■ Low  □ High  □ Unclear | ■ Low  □ High  □ Unclear | ■ Low  □ High  □ Unclear | ■ Low  □ High  □ Unclear |
| Wells (2020)^17^ | ■ Low  □ High  □ Unclear | □ Low  ■ High  □ Unclear | □ Low  ■ High  □ Unclear | ■ Low  □ High  □ Unclear | □ Low  ■ High  □ Unclear | ■ Low  □ High  □ Unclear |
| Willink (2020)^32^ | ■ Low  □ High  □ Unclear | □ Low  ■ High  □ Unclear | ■ Low  □ High  □ Unclear | ■ Low  □ High  □ Unclear | ■ Low  □ High  □ Unclear | ■ Low  □ High  □ Unclear |
| Almusawi (2021)^23^ | ■ Low  □ High  □ Unclear | ■ Low  □ High  □ Unclear | ■ Low  □ High  □ Unclear | ■ Low  □ High  □ Unclear | ■ Low  □ High  □ Unclear | ■ Low  □ High  □ Unclear |
| Tran (2021)^52^ | ■ Low  □ High  □ Unclear | ■ Low  □ High  □ Unclear | ■ Low  □ High  □ Unclear | ■ Low  □ High  □ Unclear | ■ Low  □ High  □ Unclear | ■ Low  □ High  □ Unclear |
